# Items

**Published:** 1883-02

**Authors:** 


### Items.

METEOROLOGICAL DATA.—For the month of Dec., 1882. H. C. Smyth, Observer, U. S. Signal Station, Atlanta, Ga.

| | | |
|---|---|---|
| BAROMETER —Mean | | 30.206 |
| | Highest | 30.604 on 8th. |
| | Lowest | 29.857 on 22d. |
| | Range | .747 |
| THERMOMETER—Mean | | 40.9 |
| | Highest | 64.0 on 5th. |
| | Lowest | 11.5 on 8th. |
| | Range | 52.5 |
| | Greatest Daily Range | 39.0 on 7th. |
| | Least Daily Range | 5.5 on 20th. |
| Humidity—Mean | | 67.1 |
| Dew-point—Mean | | 30.0 |
| Rainfall—Total | | 4.37 inches. |
| | Greatest Daily | 1.30 on 20th. |
| | Total Number of Days | 14 |
| Wind—Prevailing Direction | | N. W. |
| | Maximum Velocity | E., on 20th, 37 miles. |

---

IN the death of Dr. John Forsyth Meigs, which occurred December 16th, 1882, Philadelphia has sustained the loss of one of her most eminent and accomplished physicians. In commenting upon his life, work and death the *Medical News* says: "The sadness inspired by the death of Dr.

Meigs is not mitigated by the knowledge that he has fallen a victim to that peculiarly American habit of life in which a maximum of labor is associated with a minimum of recreation, and whose object is too often attained when the capacity of enjoying it is exhausted."

---

THE "Bellevue Medical College," of Massachusetts, supplies the basis for the latest medical education scandal.

This concern holds its charter under a statute providing for *manufacturing* and other corporations. Undisputed evidence showed that its "diplomas" had been bestowed upon grossly incompetent persons. Thus we have but another link in the chain of industrious medical diploma mills.

Patience—patience and long suffering characterize the people.

---

THE many friends of Dr. Jno. Thad. Johnson will be pained to learn that he has been lying seriously ill, at his home in this city, for several weeks past, from a severe attack of spinal congestion.

Certain groups of muscles upon the anterior part of the leg and upon the dorsal aspect of the fore-arm have been paralyzed.

For the last few days, however, there has been a general amelioration in his condition, and confident hopes of his complete recovery are now entertained.

---

THE grand secret of success in any calling is to be found in the constant exercise of three qualities—fidelity, industry and perseverence.

---

THE man who is always true to himself can never be false to others.

